# Treatment outcomes and clinical characteristics in diabetic versus non-diabetic sinonasal tuberculosis

**DOI:** 10.6026/973206300220206

**Published:** 2026-01-31

**Authors:** Praveen Kumar Tagore, Mahendra Kumar Bharti, Manish Kumar Sachan

**Affiliations:** 1 Department of General Medicine, Government Medical College, Datia, Madhya Pradesh, India; 2 Department of TB & Chest, Government Medical College, Datia, Madhya Pradesh, India; 3 Department of Ear Nose Throat, Government Medical College, Datia, Madhya Pradesh, India

**Keywords:** Sinonasal tuberculosis, diabetes mellitus, extrapulmonary tuberculosis, treatment outcomes, drug resistance, clinical profile

## Abstract

Sinonasal tuberculosis (SNTB) is a rare extrapulmonary presentation with poorly understood interplay with diabetes mellitus (DM). Therefore, it is of interest to compare the clinical presentation, microbiological characteristics and treatment outcomes between 60 patients with DM with SNTB and 60 non-diabetic controls treated in one of the tertiary referral centers. DM patients had a longer duration of symptoms (18.4±6.2 vs 11.2±4.1 weeks, p=0.001), more complications (41.7% vs 18.3% p=0.004) and more disease on imaging (bony involvement: 55.0% vs 31.7, p=0.011). The culture positivity (78.3 vs 58.3, p=0.015) and drug resistance (multidrug- resistance: 15.0 vs 3.3, p=0.026) were more positive in microbiologically positive patients of DM. The results of treatment showed slower conversion of sputum in DM patients (8.3±2.1 vs 6.1±1.8 weeks, p<0.001), longer therapy period (9.8±1.2 vs 8.2±0.9 months, p<0.001) and reduced success rates (78.3% vs 91.7, p=0.032). Thus, outcome success was negatively related to glycemic control (r= -0.67, p=0.001).

## Background:

Sinonasal tuberculosis (SNTB) is an uncommon, yet, clinically relevant type of extrapulmonary tuberculosis (EPTB), comprising of 1.5-5.5 percent of all EPTB cases and about 0.1 percent of all tuberculosis (TB) infections [1]. Clinical manifestations often resemble chronic rhinosinusitis, nasal polyposis or neoplastic tumors and it causes diagnostic delays of 3-6 months and inappropriate treatments such as recurrent surgical debridements [2]. The increasing burden of TB in the world, especially in the light of antimicrobial resistance and the immunocompromised groups, has intensified the requirement of increased clinical suspicion and standardized methods of managing SNTB [3]. Diabetes mellitus (DM) has become one of the most important comorbidities changing the pathophysiology and response to treatment of TB fundamentally. It is estimated that globally there are 537 million adults with DM with TB-DM comorbidity prevalence as high as 1525 percent in endemic countries [4]. Hyperglycemia inhibits the phagocytosis of macrophages, suppresses T-helper 1 cell functions and affects neutrophil chemotaxis, which leads to a permissive environment of Mycobacterium tuberculosis growth and spread [5]. Therefore, pulmonary TB in DM patients is more prevalent in terms of bacillary loads, cavitary disease and treatment failure rates than in non-diabetic patients [6].

Although there is strong evidence supporting the association of DM with deleterious pulmonary TB effects, there is no data available that is very specific to SNTB. A systematic review that was conducted in 2019 found 12 case series about SNTB with DM, all of which were descriptive and none of which could be compared by the outcome analysis [7]. Recent research in India and Brazil recorded delayed diagnosis and increased complication in DM-related SNTB, which were confined by small sample sizes (n<30) and a lack of matched controls [8, 9]. The immunopathological processes of the progression of SNTB in DM, such as the localized dysregulation of cytokines and the dysfunction of granuloma, are still conjectural and not supported by evidence [10]. Moreover, the effect of DM on SNTB microbiology, specifically smear positivity, culture recovery and drug resistance pattern, has not been explored systematically, although they are very important in managing infection and in the treatment regimen. The SNTB outcomes of treatment are traditionally projected on the basis of pulmonary TB with the general recommendation of 6-9 months of multi-drug treatment [11]. Nonetheless, delayed sputum conversion, heightened hepatotoxicity and drug-drug interactions with oral hypoglycemic drugs are common in DM patients, which require individual management [12]. The demand of DM-specific TB treatment algorithms has been outlined by the World Health Organization, but there are no prospective studies or large-scale comparative trials that support modified regimens in SNTB treatment [13]. Therefore, it is of interest to fill these gaps through an intensive comparative study of clinical profile, microbiological characteristics and treatment outcomes in DM and non-DM patients with SNTB.

## Materials and Methods:

## Study design and setting:

The case-control study was a retrospective type in which the department of Otorhinolaryngology (ENT) and General Medicine along with TB & Chest diseases Department of GMC conducted the study between January 2019 and December 2023.

## Sample size and selection of participants:

Electronic medical records were used to identify consecutive patients with SNTB. One hundred and twenty patients were recruited into the study: 60 DM (cases) and 60 controls. The reason was to determine the sample size based on the data of piloting, 80 percent power, alpha = 0.05 expecting 20 percent differentiation between the treatment success rates of each group, which gives a minimum of 54 patients per group.

## Inclusion and exclusion criteria:

Inclusion criteria included: age 18-70 years; histopathological or microbiological diagnosis of SNTB; full medical record; 12 months of follow-up data. The exclusion criteria were: HIV infection; malignancy; immunosuppressive treatment; previous anti-tubercular treatment; multidrug-resistant TB (MDR-TB) at baseline (to avoid confounding); and incomplete glycemic data in DM patients.

## Data collection variables:

Based on a standardized proforma, demographic data (age, gender, socioeconomic status), clinical issues (duration of symptoms, presenting complaints, endoscopic findings), radiology (computed tomography [CT] scan findings including bony erosion, soft tissue density), microbiological (acid-fast bacilli [AFB] smear, culture on Lowenstein-Jensen medium, GeneXpert MTB/RIF assay) and drug susceptibility testing, glycemic control (HbA1c levels at diagnosis), treatment (drug regimen, duration, adherence) and outcomes (symptom resolution, radiological improvement, microbiological conversion, complications, recurrence) were systematically recorded and analyzed.

## Diagnostic criteria:

The criteria in SNTB diagnosis were positive AFB smear/culture based on nasal tissue; histopathy with caseating granuloma and positive TB-PCR; or GeneXpert on nasal swab. Diagnosis of DM was based on WHO criteria (fasting glucose 126mg/dL, HbA1c 6.5% or history).

## Treatment protocols:

All patients underwent weight-based standard first-line treatment (isoniazid 5 mg/kg, rifampicin 10mg/kg, pyrazinamide 25mg/kg, ethambutol 15mg/kg) 2 months intensive phase and thereafter, isoniazid and rifampicin continuation. DM patients were put through simultaneous concomitant glycemic optimization (target HbA1c <7%). The course of treatment was tailored on response. All patients were introduced to direct therapy (DOTS).

## Outcome definitions:

Success in treatment was determined as the completion of therapy with clinical remission and microbiological negativity at 12 months. Failure included default of treatment, death or persistent/recurring disease. Conversion time Sputum conversion time was time taken to achieve two negative AFB smears/cultures.

## Statistical analysis:

The SPSS version 26.0 was used to analyze the data. Continuous variables were compiled in the form of Mean ± SD or median (IQR) and compared with independent t-test or Mann Whitney U test. Chi-square or Fisher exact test was applied to analyze the categorical variables. Pearson correlation was used to determine relationships between HbA1c outcomes. The independent predictors of the failure to respond to treatment were determined using multivariate logistic regression. The significance rate was established as p<0.05.

## Results:

The study included 120 patients (72 males, 48 females) with mean age 46.8±12.3 years. DM patients were older (49.2±11.8 vs 44.4±12.5 years, p=0.038) and had higher body mass index (24.6±3.8 vs 22.1±3.2 kg/m^2^, p=0.001). Symptom duration was significantly prolonged in DM patients (18.4±6.2 vs 11.2±4.1 weeks, p<0.001). Past TB contact history was comparable (DM: 31.7%, non-DM: 28.3%, p=0.678). Mean HbA1c in DM patients was 9.2±1.8% (Table 1). Presenting symptoms differed significantly: DM patients exhibited higher rates of facial swelling (45.0% vs 26.7%, p=0.031), nasal obstruction (78.3% vs 58.3%, p=0.019) and headache (63.3% vs 41.7%, p=0.016). Epistaxis was more common in non-DM patients (36.7% vs 20.0%, p=0.038). Endoscopically, DM patients demonstrated necrotic tissue (51.7% vs 30.0%, p=0.012) and septal perforation (15.0% vs 3.3%, p=0.026) more frequently. CT imaging revealed bony erosion in 55.0% of DM versus 31.7% of non-DM patients (p=0.011). Microbiologically, AFB smear positivity was lower in DM patients (43.3% vs 66.7%, p=0.009), but culture positivity was higher (78.3% vs 58.3%, p=0.015). GeneXpert detected rifampicin resistance in 18.3% of DM versus 6.7% of non-DM patients (p=0.045) (Table 2). Sputum conversion time was significantly delayed in DM patients (8.3±2.1 vs 6.1±1.8 weeks, p<0.001). Treatment duration averaged 9.8±1.2 months in DM versus 8.2±0.9 months in non-DM patients (p<0.001). Complications occurred in 41.7% of DM versus 18.3% of non-DM patients (p=0.004), predominantly sinusitis (23.3% vs 10.0%) and osteomyelitis (11.7% vs 3.3%). Treatment success rates were lower in DM patients (78.3% vs 91.7%, p=0.032). Default rates were higher in DM (13.3% vs 5.0%, p=0.089, borderline). HbA1c levels correlated inversely with treatment success (r=-0.67, p<0.001) (Table 3).

## Discussion:

This retrospective case-control study has shown significant differences in clinical presentation, microbiological features and treatment outcomes in diabetic and non-diabetic patients with sinonasal tuberculosis. The results highlight the importance of DM as a key determinant of the course of SNTB disease, which presents itself in the form of late diagnosis, an increased rate of tissue damage, a change in bacteriological composition and deterioration in therapeutic outcomes. The prolonged period of symptoms (18.4 weeks) in DM patients is due to the problem of immunopathological changes as well as diagnostic difficulties. Hyperglycemia disrupts an early inflammatory reaction and may conceal symptoms of TB, permitting a slow progression of the disease [14]. This late onset is associated with late radiological appearances that were demonstrated by half of the DM patients develop 55% bony erosion compared to just 32% in controls. These destructive phenotypes are consistent with the research on tuberculous osteomyelitis, during which DM increases bone resorption by promoting the activation of osteoclasts mediated by advanced glycation end-products [15]. The increased incidence of necrotic tissue and septal perforation further suggest vicious local pathology, which is probably aggravated by microvascular insult and wound healing impairment inherent in DM [16].

The results of microbiological tests demonstrated a contradictory trend, which was the absence of smear positivity but an increase in culture positivity in DM patients. This can be an indication of variants of paucibacillary disease with changed bacterial shedding that has already been observed in diabetic pulmonary TB whereby the sensitivity of the sputum smear reduces by 15-20 percent [6]. Higher culture yields on the other hand suggest that at the time of infection, bacillary load in tissue is large, perhaps because of the impaired containment. The result of 18.3 percent resistance to rifampicin in DM patients is alarming as it is much higher than the control and global rates of 6.7 percent and 3 to 4 percent of new TB cases respectively [17]. It could be due to the previous accidental exposure to antibiotics in DM patients with recurrent infections, or pharmacologic changes where poor absorption of the drug as a result of diabetic gastropathy promotes selection of resistance [12]. The outcomes of treatment are unambiguous DM-related harm. The 8.3-week sputum conversion period is higher than the 6.1 weeks of controls and normal pulmonary TB references, which are supported by meta-analyses of 1.5 years longer DM-TB conversion [18]. This latency puts the infection control at risk and exposes the transmission to danger. The 9.8-month treatment period that was empirically lengthened by clinicians yet provided 21.7% failure rates was a pale shadow of the 8.3% in non-DM patients. This is consistent with literature of 23 fold increased failure rates in DM-related EPTB which were explained by insufficient levels of drugs and immune impairment [8]. The negative correlation between the HbA1c and outcome (r= -0.67) strengthens the idea that glycemic control is a determinant of outcome that is modifiable and in our regression equation; an increase of 1% in HbA1c decreases the probability of success by 18%. The complication rate of DM patients of 41.7 percent (majority being sinuusitis and osteomyelitis) indicates the necessity of intensive adjunctive care. The dysfunction of leukocyte and hyperglycemia preconditions the secondary bacterial infection and impaired healing of the bone [19]. Septal perforation, which is three times more common in DM patients, is a structural sequela of permanent nature and requires complicated reconstruction, highlighting the functional morbidity in addition to resolution of the infection.

We differ with the results of other sources on pulmonary TB in which there are less of DM effects. This possibly indicates the special immunology of sinonasal mucosa, with local IgA responses and mucociliary clearance, both impaired in DM, having central roles [20]. NTB sequestration into the anatomy can also affect the penetration of drugs, which may worsen the inherent pharmacokinetics of DM [11]. The strengths of the study design are a relatively big sample size, a rigid exclusion of confounding factors such as HIV and thorough evaluation of the outcomes. The matched control case-control design increases comparability. Nonetheless, restrictions deserve to be mentioned. Retrospective design presents possibilities of selection and information bias but this was curbed by standardized data collection. The recruitment through a single center was in a tertiary hospital, which might not be generalizable to the community context. The single time-point measurements of HbA1c compared to longitudinal glycemic trends, which are potentially more accurate predictors, were used. Also, there was no level of monitoring of the drug levels thereby not allowing a conclusive attribution of the failure of treatment to be due to pharmacokinetic versus immunological influence. These results should be prospectively confirmed in a number of centers in future research to include therapeutic drug monitoring and repeated HbA1c tests. Extended-period (12 months) and/or increased doses of rifampicin in DM-SNTB should be assessed in randomized trials. Mechanistic pathways could be clarified by immunophenotyping studies of nasal mucokal cytokine pattern (TNF-alpha, IFN- gamma, IL- 10) [21]. Lastly, the policy of the routine DM screening of SNTB patients would be informed by the cost-effectiveness analysis. Finally, this paper confirms DM as a significant factor of unfavorable outcomes in SNTB, which is exhibited by delayed presentation, changed microbiology and therapeutic refractory. Clinicians should have high levels of suspicion to SNTB among diabetic patients with refractory sinonasal symptoms, pursue aggressive tissue diagnosis and focus on glycemic optimization as an inseparable part of TB therapy. SM regimens might be inadequate in DM patients and their extension and close observation should be considered.

## Conclusion:

We show that patients with diabetes mellitus and sinonasal tuberculosis have a different clinical picture because of long periods of symptoms, active tissue destruction, low smear positivity, high culture positivity and low rates of drug resistance in comparison with non-diabetic controls. The result of the treatment is significantly impaired and the conversion of sputum is delayed, the treatment process prolongs, the number of complications and success rates reduces. These results support the use of combined management guidelines that include screening of DM early, strict glycemic control, prolonged use of anti-tubercular therapy and close monitoring of complications among high-risk populations.

## Figures and Tables

**Table 1:** Baseline demographic and clinical characteristics

**Characteristic**	**DM Group (n=60)**	**Non-DM Group (n=60)**	**p-value**
Age (years), Mean±SD	49.2±11.8	44.4±12.5	0.038
Gender (male/female)	38/22	34/26	0.456
BMI (kg/m^2^), Mean±SD	24.6±3.8	22.1±3.2	0.001
Symptom duration (weeks), Mean±SD	18.4±6.2	11.2±4.1	<0.001
Past TB contact, n (%)	19 (31.7)	17 (28.3)	0.678
HbA1c (%), Mean±SD	9.2±1.8	5.4±0.5	<0.001
Smoking history, n (%)	16 (26.7)	14 (23.3)	0.664
SD: Standard Deviation;
BMI: Body Mass Index;
HbA1c: Glycated Hemoglobin

**Table 2:** Clinical presentation and microbiological profile

**Parameter**	**DM Group (n=60)**	**Non-DM Group (n=60)**	**p-value**
**Symptoms, n (%)**			
Facial swelling	27 (45.0)	16 (26.7)	0.031
Nasal obstruction	47 (78.3)	35 (58.3)	0.019
Headache	38 (63.3)	25 (41.7)	0.016
Epistaxis	12 (20.0)	22 (36.7)	0.038
**Endoscopic findings, n (%)**			
Necrotic tissue	31 (51.7)	18 (30.0)	0.012
Septal perforation	9 (15.0)	2 (3.3)	0.026
**CT findings, n (%)**			
Bony erosion	33 (55.0)	19 (31.7)	0.011
Soft tissue density	52 (86.7)	48 (80.0)	0.312
**Microbiology, n (%)**			
AFB smear positive	26 (43.3)	40 (66.7)	0.009
Culture positive	47 (78.3)	35 (58.3)	0.015
GeneXpert positive	51 (85.0)	54 (90.0)	0.417
Rifampicin resistance	11 (18.3)	4 (6.7)	0.045
AFB: Acid-Fast Bacilli;
CT: Computed Tomography

**Table 3:** Treatment outcomes and complications

**Outcome**	**DM Group (n=60)**	**Non-DM Group (n=60)**	**p-value**
Sputum conversion (weeks), Mean±SD	8.3±2.1	6.1±1.8	<0.001
Treatment duration (months), Mean±SD	9.8±1.2	8.2±0.9	<0.001
Complications, n (%)	25 (41.7)	11 (18.3)	0.004
Sinusitis	14 (23.3)	6 (10.0)	0.036
Osteomyelitis	7 (11.7)	2 (3.3)	0.067
Orbital involvement	4 (6.7)	3 (5.0)	0.701
Treatment success, n (%)	47 (78.3)	55 (91.7)	0.032
Treatment failure, n (%)	13 (21.7)	5 (8.3)	0.032
Default	8 (13.3)	3 (5.0)	0.089
Death	2 (3.3)	1 (1.7)	0.558
Persistent disease	3 (5.0)	1 (1.7)	0.618
Multivariate logistic regression identified
HbA1c >8% (OR=3.87, 95% CI: 1.62-9.24, p=0.002) and
presence of bony erosion (OR=2.94, 95% CI: 1.18-7.31,
p=0.020) as independent predictors of treatment failure.
